# Program and Faculty Reputation Are Valued Most by Applicants to Orthopaedic Sports Medicine Fellowships

**DOI:** 10.1016/j.asmr.2022.10.014

**Published:** 2022-12-14

**Authors:** Elisabeth H. Geraghty, Matthew J. Kraeutler, Sean C. Clark, Eric C. McCarty, Mary K. Mulcahey

**Affiliations:** a Department of Orthopaedics, University of Colorado School of Medicine, Aurora, Colorado; b Department of Orthopedics & Sports Medicine, Houston Methodist Hospital, Houston, Texas; c Department of Orthopaedic Surgery, Tulane University School of Medicine, New Orleans, Louisiana, U.S.A.

## Abstract

**Purpose:**

To determine the top orthopaedic surgery sports medicine fellowship programs in the United States and the most important aspects of fellowship programs as perceived by applicants.

**Methods:**

An anonymous survey was distributed via e-mail and text message to all current/former orthopaedic surgery residents who applied to one particular orthopaedic sports medicine fellowship program during the 2017-2018 through 2021-2022 application cycles. The survey asked applicants to rank what they considered to be the top-10 orthopaedic sports medicine fellowship programs in the United States before and after completion of their application cycle, based on operative and nonoperative experience, faculty, game coverage, research, and work–life balance. Final rank was calculated by awarding 10 points for a first-place vote, 9 points for a second-place vote, etc., with total number of points used to determine final ranking for each program. Secondary outcomes included rates of applying to perceived top-10 programs, relative importance of different fellowship program aspects, and preferred type of practice.

**Results:**

Seven-hundred sixty-one surveys were distributed with 107 applicants responding (14% response rate). Applicants voted the top orthopaedic sports medicine fellowships programs to be: (1) Steadman Philippon Research Institute, (2) Rush University Medical Center, and (3) Hospital for Special Surgery, both before and following the application cycle. When ranking fellowship program aspects, faculty members and fellowship reputation were most likely to be ranked highest in importance.

**Conclusions:**

This study demonstrates that most orthopaedic sports medicine fellowship applicants highly valued program reputation and faculty members when choosing a fellowship program and that the application/interview process did not have a substantial effect on how individuals perceived the top programs.

**Clinical Relevance:**

The findings of this study are important for residents applying to orthopaedic sports medicine fellowships and may have implications on fellowship programs and future application cycles.

Orthopaedic surgery is one of the most competitive fields in medicine, with a match rate of approximately 67%.^1^ Over the past 2 decades, there has been a significant trend toward fellowship training and subspecialization in orthopaedic surgery in the United States.^2^, ^3^, ^4^, ^5^, ^6^ Due to the advancements and variety of subspecialties within orthopaedic surgery, many residents pursue fellowships to learn advanced techniques, difficult procedures, and improve job opportunities.^1^^,^^7^ Of orthopaedic fellowships, sports medicine is the most popular subspecialty choice, with the highest number of fellowship positions.^8^, ^9^, ^10^ As a consequence of its popularity, sports medicine fellowship programs are extremely competitive. Orthopaedic residents apply to a significantly greater number of sports medicine fellowship programs than other subspecialties.^11^, ^12^, ^13^ With the coronavirus disease 2019 pandemic, this competition was amplified by the introduction of virtual fellowship interviews, which allowed residents the freedom to apply to more programs and “attend” more interviews.^14^^,^^15^ Despite this, sports medicine fellowships have excellent match characteristics, with nearly 50% of applicants matching into their #1 ranked program.^10^^,^^12^^,^^16^

Several studies have investigated factors that orthopaedic fellowship program directors consider important when ranking applicants.^11^^,^^17^, ^18^, ^19^ However, despite the high number of orthopaedic sports medicine fellowship applicants and growing interest in the field, fellowship applicants’ attitudes and preferences toward programs are not well understood. The purpose of this study was to determine the top orthopaedic surgery sports medicine fellowship programs in the United States and the most important aspects of fellowship programs as perceived by applicants. The authors hypothesized that there would be substantial agreement across former applicants regarding the top orthopaedic sports medicine fellowship programs.

## Methods

Following approval from the institutional review board at the senior author’s (M.K.M) institution, an anonymous electronic survey was distributed via email in March and April of 2022 to all individuals who applied to one specific orthopaedic sports medicine fellowship program during the 2017-2018 through 2021-2022 application cycles. This date range provided an adequate sample size and was not expanded beyond 5 applicant cycles to reduce applicant recall bias and reflect current applicant rankings and application patterns. Applicants from the 2021-2022 cycle were contacted following the Match in April 2022. For individuals whose listed e-mail no longer worked, the survey was distributed via text message. After initial distribution, a follow-up e-mail/text message was distributed at 2 and 4 weeks to increase participation. The survey consisted of 13 questions (Appendix Table 1, available at www.arthroscopyjournal.org). The survey asked applicants to rank what they considered to be the top-10 orthopaedic sports medicine fellowship programs in the United States before and after completion of their application cycle, based on operative and nonoperative experience, faculty, game coverage, research, and work–life balance. The survey included all 89 orthopaedic sports medicine fellowships accredited by the Accreditation Council for Graduate Medical Education during the 2021-2022 application cycle. Final rank was calculated by awarding 10 points for a first-place vote, 9 points for a second-place vote, etc., with total number of points used to determine final ranking for each program. The total number of top-10 votes and average rating of each program were included as additional measures of perceived rank. Average rating was calculated by averaging the top-10 vote ranks each program received. A lower average rating indicates a program received higher rankings. Although respondents were only asked to rank their top-10 programs, the final rankings of the top-20 programs before and upon completion of the application cycle were calculated. Secondary outcomes included rates of applying to perceived top-10 programs, relative importance of different fellowship program aspects, and preferred type of practice. Descriptive statistics were used to analyze the data. Average ranking of fellowship program aspects was calculated as a weighted average of the applicants’ responses, where “1” was the most important and “9” was the least important.

## Results

Seven-hundred sixty-one surveys were distributed with 107 applicants responding (14% response rate). Most of the respondents were applicants from the 2020-2021 cycle (35%). The respondents’ residency programs represented a relatively even geographic distribution across the 4 regions with slightly more respondents coming from the Northeast (n = 21, 29%) and Midwest (n = 24, 32%). Nearly 70% of applicants (n = 51) were aged 31-35 years (Table 1). Ninety percent of respondents were male (n = 66). The majority of respondents were White (n = 63, 85.1%).

**Table 1:** Demographic Information of the Survey Respondents

Demographics	Count	Percentage
Application cycle		
2017-2018	26	25.2%
2018-2019	12	11.7%
2019-2020	23	22.3%
2020-2021	36	35.0%
2021-2022	6	5.8%
Region		
Northeast	21	28.8%
Midwest	24	32.9%
South	14	19.2%
West	14	19.2%
Age, y		
<25	0	0.0%
26-30	2	2.7%
31-35	51	69.9%
36-40	17	23.3%
41-45	3	4.1%
46-50	0	0.0%
>50	0	0.0%
Sex		
Male	66	90.4%
Female	7	9.6%
Transgender male	0	0.0%
Transgender female	0	0.0%
Gender binary nonconforming	0	0.0%
Other	0	0.0%
Prefer not to say	0	0.0%
Race/ethnicity		
White	63	85.1%
Asian	7	9.5%
Native Hawaiian or Pacific Islander	1	1.4%
Other	3	4.1%
Black or African American	0	0.0%
Hispanic, Latino, or Spanish	0	0.0%
American Indian or Alaska Native	0	0.0%

When asked to rank what they considered to be the top-10 orthopaedic sports medicine fellowship programs in the United States before their application cycle, applicants voted the top programs to be (1) Steadman Philippon Research Institute (SPRI), (2) Rush University Medical Center (RUMC), and (3) Hospital for Special Surgery (HSS) (Table 2). SPRI received 71 top-10 votes (71/107, 66%) and had an average rating of 2.65 (±2.09). RUMC had 72 top-10 votes (72/107, 67%), but a slightly lower average rating of 3.26 (±2.62). HSS received 66 top-10 votes (66/107, 62%), with an average rating of 3.80 (±2.47).

**Table 2:** The Top-20 Orthopaedic Sports Medicine Fellowship Programs as Ranked by Fellowship Applicants Before Completion of Their Application Cycle

Before Application Cycle				
Rank	Program	No. Top 10 Votes	Avg. Rating	Score
1	Steadman Philippon Research Institute Program	71	2.65	593
2	Rush University Medical Center Program	72	3.26	557
3	Hospital for Special Surgery/Cornell Medical Center Program	66	3.8	475
4	Cedars-Sinai Kerlan-Jobe Orthopaedic Clinic Program	56	4.87	343
5	OrthoCarolina Sports Medicine, Shoulder & Elbow Program	43	5.28	246
6	American Sports Medicine Institute (St. Vincent’s) Program	36	5.01	216
7	University of Pittsburgh/UPMC Medical Education Program	41	6.05	203
8	Steadman Hawkins Clinic - Denver Program	30	4.67	190
9	Duke University Hospital Program	38	6.52	175
10	Steadman Hawkins Clinic of the Carolinas Program	27	5.15	158
11	Stanford Orthopaedic Sports Medicine Fellowship Program	27	6.7	116
12	University of Utah Program	22	6.05	109
13	Andrews Research and Education Foundation	22	6.32	103
14	Mayo Clinic (Rochester), College of Medicine Program	20	6.05	99
15	Mississippi Sports Medicine & Orthopaedic Center Program	18	5.78	94
16	University of Virginia Program	21	6.76	89
17	University of Connecticut Program	17	6.06	84
18	Massachusetts General Hospital/Harvard Medical School Program	17	6.53	76
19	University of California San Francisco Program	17	6.59	75
20 (tie)	Cleveland Clinic Foundation Sports Medicine Program	19	7.62	62
20 (tie)	University of Colorado Health Sciences Center Program	17	7.35	62

NOTE. Ranks were determined by total score.

When asked to rank the top-10 orthopaedic sports medicine fellowship programs in the United States upon completion of their application cycle, applicants still voted the top orthopaedic sports medicine fellowship programs to be: (1) SPRI, (2) RUMC, and (3) HSS (Table 3). There was a 33% decrease in voting for this question (only 592 votes vs 878 initially), so a natural decrease in number of top-10 votes and therefore total score for each program was expected. The top 10 programs remained the same, but with a slightly different order (Table 3). Eighteen of the top-20 programs remained in the top-20 upon completion of the application cycle. Voting remained relatively stable, as the top-10 programs had 54.7% of the votes before the application cycle and had 51.2% of the votes after the application cycle. Despite a decrease in voting for this question, 21 programs gained top-10 votes after completion of the application cycle.

**Table 3:** The Top-20 Orthopaedic Sports Medicine Fellowship Programs as Ranked by Fellowship Applicants Upon Completion of Their Application Cycle

Upon Completion of Application Cycle				
Rank	Program	No. Top 10 Votes	Avg. Rating	Score
1	Steadman Philippon Research Institute Program	43	3.35	329
2	Rush University Medical Center Program	45	4.11	310
3	Hospital for Special Surgery/Cornell Medical Center Program	41	4.71	258
4	OrthoCarolina Sports Medicine, Shoulder & Elbow Program	31	5.55	169
5	Cedars-Sinai Kerlan-Jobe Orthopaedic Clinic Program	30	5.4	168
6	American Sports Medicine Institute (St. Vincent's) Program	25	4.56	161
7	University of Pittsburgh/UPMC Medical Education Program	24	5.25	138
8	Steadman Hawkins Clinic of the Carolinas Program	21	4.64	132
9	Steadman Hawkins Clinic - Denver Program	19	4.21	129
10	Duke University Hospital Program	24	6.54	107
11	University of Utah Program	16	4.81	99
12	University of Colorado Health Sciences Center Program	14	4.21	95
13	Mississippi Sports Medicine & Orthopaedic Center Program	15	5.13	88
14	University of Virginia Program	16	6.5	72
15	University of Connecticut Program	14	6.29	66
16	Stanford Orthopaedic Sports Medicine Fellowship Program	14	6.5	63
17	University of California San Francisco Program	10	5.7	53
18	Mayo Clinic (Rochester), College of Medicine Program	10	7.5	35
19	Cleveland Clinic Foundation Sports Medicine Program	9	7.22	34
20 (tie)	The University of Texas Health Science Center at Houston Sports Medicine	8	6.87	33
20 (tie)	NYU Hospital for Joint Diseases	8	6.87	33

NOTE. Ranks were determined by total score.

Forty-three percent of applicants (43/99) applied to all of their perceived top-10 programs, with 67% of respondents (66/99) applying to at least 8 of their top-10 programs. Interestingly, 13 of 99 applicants (13%) applied to 4 or fewer of their top-10 programs. Of those who did not apply to all programs in their top-10, 43 of 46 (94%) applicants thought the program did not fit their preferences, and 21 of 46 (46%) thought they were not competitive for those programs. Nearly all of the applicants agreed/strongly agreed (70/71, 99%) that the personalities of the attendings they met on their interviews influenced their rank list decision upon completion of the application cycle. When ranking fellowship program aspects, faculty members (average rating of 2.54 ± 1.51) and fellowship reputation (2.69 ± 1.84) were most likely to be ranked highest in importance (Table 4). Most applicants (62/74, 84%) applied to both academic and private practice programs. Interestingly, only 1 of 74 (1.4%) applicants applied exclusively to private practice programs, even though nearly 40% (29/73) wanted to or did go into private practice. Behind private practice, the next most popular options that applicants wanted to join or did join after fellowship were hospital-employed (n = 16, 21.9%) and academic medical centers (n = 15, 20.6%).

**Table 4:** Average Rankings of the Factors Considered Most Important by Applicants When Deciding to Which Fellowship Programs to Apply

Factors Considered Most Important by Sports Medicine Fellowship Applicants	Average Ranking
Faculty members	2.54
Reputation	2.69
Specific procedures performed	3.2
Location	3.8
Sports team coverage opportunities	4.97
Number of fellows	6.24
Private practice	6.5
Research opportunities	6.72
Academic hospital	7.54

NOTE. Average ranking is calculated as a weighted average of the applicants’ responses (“1” indicates the most important criterion; “9” indicates the least important criterion).

## Discussion

Based on the results of our study, orthopaedic sports medicine fellowship applicants voted the top fellowship programs to be (1) SPRI, (2) RUMC, and (3) HSS, both before and following the application cycle. When asked to rank programs upon completion of the application cycle, each of the top-10 programs before the application cycle remained in the top-10 and 18 of the top-20 programs remained in the top-20. These findings suggest the application process does not have a substantial effect on how applicants perceive the top programs. When ranking fellowship program aspects, faculty members and fellowship reputation were most likely to be ranked highest in importance.

Sports medicine is the most competitive subspecialty of the orthopaedic surgery fellowships, and these applicant rankings may give future applicants insight into which programs are considered top tier. From 1984 to 2014, orthopaedic job openings requiring fellowship training increased from 5% to 68%.^3^ In addition, sports medicine has the highest number of fellows per advertised jobs (6.3) of all orthopaedic subspecialities.^20^ Completing fellowships at top-tier programs will likely give graduates an advantage by making them more competitive for job opportunities in the future. For example, one study found that a majority of orthopaedic shoulder and elbow fellows believed completing a fellowship enhanced their job opportunities.^21^

Our study demonstrated that orthopaedic sports medicine fellowship applicants find faculty members and fellowship reputation to be the most important factors when deciding to which programs to apply. This finding is supported by previous studies,^5^^,^^13^ which found that orthopaedic surgery residents applying to fellowship programs value faculty and staff member personalities more than other factors when choosing a program. Previous literature regarding the importance of program reputation to applicants has provided mixed conclusions. In contrast with our findings, Zeoli et al.^13^ reported that although many orthopaedic sports medicine fellowship applicants ranked program reputation as very/extremely important, it was not considered a top 3 factor when forming an impression of a program. In 2021, Oser et al.^22^ found that sports medicine fellowship applicants viewed reputation of faculty members as one of the most important factors whereas program prestige was ranked with moderate importance. Li et al.^9^ reported that orthopaedic fellowship applicants of all subspecialities ranked overall program reputation as more important than faculty and staff reputation. In our study, “reputation” was listed as one factor and was not segregated into program and faculty reputations. It is possible that survey respondents viewed reputation as more important in our study because they interpreted reputation to mean both overall program and faculty reputation. Interestingly, the majority of research shows that fellowship applicants find surgical case volume/complexity/variety to be the most important factor when ranking programs.^5^^,^^9^^,^^13^^,^^22^ Although “surgical volume and variety” was not a listed factor in our survey, the respondents in our study found “specific procedures performed” to be the third-most important factor.

It has been previously reported that orthopaedic residents place less value on research opportunities when ranking orthopaedic surgery fellowships.^5^ Our study demonstrated that sports medicine fellowship applicants find research opportunities less important when ranking programs. Despite this minimal interest in research opportunities, there has been an overall increase in the number of publications among sports medicine fellowship applicants over the last several academic years.^23^ This would suggest that applicants might not actually value the research itself, but instead perform research to improve their competitiveness as an applicant. Mayfield et al.^24^ sought to rank orthopaedic sports medicine fellowships in the United States based on academic productivity. The authors found the top-5 programs to be (1) HSS, (2) RUMC, (3) University of Pittsburgh Medical Center (UPMC), (4) Mayo Clinic (Rochester), and (5) Boston Children’s Hospital. Of interest, 5 of the top-10 academic programs from this study were also included on the top-10 list from our study. Thus, although not all applicants weigh research opportunities when ranking programs, they often find program reputation to be an important factor and programs gain reputation through their academic impact.

Our study found faculty member interactions to be one of the most important factors to applicants when ranking a fellowship program and, unfortunately, virtual interviews offer limited opportunities to engage with faculty. A recent study by Clark et al.^14^ found that 43% of applicants thought virtual interviews negatively affected their personal connections with the fellowship program. Given the elimination of the financial and time burden associated with interview travel over the last 2 application cycles with virtual interviews, it is possible that this method of fellowship interviews could emerge as the new standard.^15^^,^^25^ With virtual interviews, residents can participate in more interviews, minimize time away from residency training, and save upwards of $5,000 or more on travel. Consequently, both program directors and applicants are in favor of having the option to interview virtually, which suggests they will likely continue to play a role in future application cycles.^14^^,^^15^^,^^25^ However, as a result of the limited personal interactions afforded by virtual interviews, it is often difficult for applicants to assess a program’s “feel,” sense of community, nonstaged interactions, and life outside of the hospital setting.^15^ As a result, residents may need other ways, such as social media and fellowship program websites, to learn about these programs without being able to see them and meet faculty in-person. One potential solution is the creation of unofficial applicant rankings such as those listed in this study.

### Limitations

There are several limitations to this study. Our survey had a relatively low response rate and, therefore, the results may not reflect the opinions of all current/previous orthopaedic sports medicine fellowship applicants. The survey was sent to all applicants of one particular fellowship program over the last 5 application cycles, and therefore there may be bias based on the location or other aspects of this program. Response bias plays a role in this study, as applicants who matched to fellowship programs not typically perceived as a top program may have been less likely to complete the survey. Also, this survey may be impacted by recall bias as applicants might incorrectly remember their exact attitudes and rank lists before and after the application cycle. Not every respondent completed all of the survey questions. Lastly, the list of important fellowship aspects was not exhaustive and may not have included every aspect of the sports medicine fellowship application process that applicants find important. Applicants may also rank factors differently depending on how they were interpreted.

## Conclusions

This study demonstrates that most orthopaedic sports medicine fellowship applicants highly valued program reputation and faculty members when choosing a fellowship program and that the application/interview process did not have a substantial effect on how individuals perceived the top programs.

## Figures and Tables

**Appendix Table 1:** Complete List of Survey Questions

Q1. In which application cycle did you participate?
Responses: “2017-2018”, “2018-2019”, “2019-2020”, “2020-2021”, “2021-2022”
Q2. Prior to completion of the application cycle, what did you consider to be the top-10 orthopaedic sports medicine fellowship programs in the United States, based on operative and nonoperative experience, faculty, game coverage, research, and work/life balance? Please rank your top-10 programs, with 1 being the best.
Responses: N/A, 1, 2, 3, 4, 5, 6, 7, 8, 9, 10 for each ACGME accredited orthopaedic sports medicine fellowship program
Q3. How many programs did you apply to in your top-10 list?
Responses: 0, 1, 2, 3, 4, 5, 6, 7, 8, 9 ,10
Q3a. Why did you not apply to all of the programs in your top-10 list?
Responses: “Programs did not fit my preferences”, “I did not think I was competitive for these programs”, “Both”
Q4. Upon completion of the application cycle, what did you consider to be the top-10 orthopaedic sports medicine fellowship programs in the United States, based on operative and nonoperative experience, faculty, game coverage, research, and work/life balance? Please rank your top-10 programs, with 1 being the best.
Responses: N/A, 1, 2, 3, 4, 5, 6, 7, 8, 9, 10 for each ACGME accredited orthopaedic sports medicine fellowship program
Q5. The personalities of the attendings I met on my interviews influenced my rank list decisions.
Responses: “Strongly Agree”, “Agree”, “Neutral”, “Disagree”, “Strongly Disagree”
Q6. Rank the following aspects of a fellowship program that were most important to you when deciding which programs to apply to with 1 being the most important and 9 being the least important.
Responses: “Reputation”, “Location”, “Specific Procedures Performed”, “Faculty Members”, “Sports Team Coverage Opportunities”, “Research Opportunities”, “Private Practice”, “Academic Hospital”, “Number of Fellows”
Q7. Did you apply to programs at an academic medical center or private practice institution?
Responses: “Academic Medical Center”, “Private Practice”, “Both”
Q8. Which type of practice do you want to join, or did you join after you have completed your fellowship?
Responses: “Academic Medical Center”, “Private Practice”, “Academic Medical Center/Private Practice Blend ‘Privademic’”, “Hospital-Employed”, “Other (please specify)”
Q9. In which region of the country is your residency program located?
Responses: “Northeast – CT, MA, NH, NJ, NY, PA, RI, VT”, “Midwest – IN, IL, IA, KS, MI, MN, MO, NE, ND, OH, WI”, “South – AL, AR, DC, KY, LA, MD, MS, NC, OK, SC, TN, TX, VA, WV”, “West – AZ, CA, CO, HI, NV, NM, OR, UT, WA”
Q10. What is your age?
Responses: “<25”, “26-30”, “31-35”, “36-40”, “41-45”, 46-50”, “>50”
Q11. With which sex do you most identify?
Responses: “Male”, “Female”, “Transgender Male”, “Transgender Female”, “Gender Binary Non-Conforming”, “Other”, “Prefer Not to Say”
Q12. Are you of Hispanic, Latino, or Spanish origin?
Responses: “Yes”, “No”
Q13. How would yo u describe yourself? (Check all that apply)
Responses: “White”, “Black or African American”, “American Indian or Alaska Native”, “Asian”, “Native Hawaiian or Pacific Islander”, “Other”

## Supplementary Data

ICMJE author disclosure forms
